# Secular Trends of Acute Viral Hepatitis Incidence and Mortality in China, 1990 to 2019 and Its Prediction to 2030: The Global Burden of Disease Study 2019

**DOI:** 10.3389/fmed.2022.842088

**Published:** 2022-03-11

**Authors:** Xing Su, Lin Zheng, Huami Zhang, Ting Shen, Yingna Liu, Xiaowei Hu

**Affiliations:** Xihu District Center for Disease Control and Prevention, Hangzhou, China

**Keywords:** trends, China, viral hepatitis, prediction, epidemiology

## Abstract

**Background:**

Understanding the patterns and trends in the context of both incidence and mortality and anticipating future trends is important for viral hepatitis prevention, treatment, and guiding resource allocation in China. The objective of this study is to provide a comprehensive temporal analysis of acute viral hepatitis and its type using the most updated data from the Global Burden of Diseases, Injuries, and Risk Factors Study (GBD 2019) to estimate the incidence and mortality of hepatitis from 1990 to 2019 and make predictions to 2030.

**Methods:**

The age-standardized incidence (ASIR) and mortality rate (ASMR) of viral hepatitis in China were obtained from the Global Burden of Diseases, Injuries, and Risk Factors Study (GBD 2019). Trends of ASIR and ASMR for viral hepatitis were plotted using locally weighted regression (LOESS). We used joinpoint regression analysis to detect temporal changes and estimate the annual percent of change (APC) of each trend segment and the corresponding 95% confidence interval (CI). A Bayesian age-period-cohort analysis was employed to describe ASIR and ASMR trends between 1990 and 2019 and projections to 2030.

**Results:**

In 1990, there were 67 million incident cases of acute viral hepatitis, which then decreased to 47 million incidence cases in 2019. Hepatitis A and hepatitis B account for the majority of acute viral hepatitis, and the most pronounced declines in hepatitis B (−48.7%) and hepatitis C (−39.0%) were observed between 1990 and 2019. The ASIR of overall acute viral hepatitis shows a persistent decline, with an average annual percent of change (AAPC) of −1.9% (95% CI: −1.9, −1.8) between 1990 and 2019. The trend of ASMR demonstrated a rapid decline between 1990 and 2005, followed by a slow decline until 2030.

**Conclusion:**

Our study reveals favorable declining trends of incidence and mortality for acute viral hepatitis in China from 1990 and 2019, and these favorable trends are predicted to continue up to 2030. Despite the favorable trends observed, the absolute number of viral hepatitis, especially hepatitis A and B, is still substantial in China. A scaled-up vaccine campaign is still needed to tackle the large number of vaccine preventable hepatitis infections.

## Introduction

Viral hepatitis is a group of diseases characterized by inflammation of the liver caused by viral infection, including acute forms that can manifest as recent infection with rapid onset, as well as chronic infections. Chronic hepatitis can lead to complications such as cirrhosis, liver failure, or even liver cancer (1). The mode of transmission varies depending on the type of viral hepatitis, with hepatitis A and E transmitted primarily by the fecal-oral route and hepatitis B, C, and D transmitted by contact with infectious blood or body fluids. Viral hepatitis is an international public threat in almost all countries (2), and China is probably the most affected country (3, 4), accounting for one-third of hepatitis B and 7% of hepatitis C global infections (5). Given its substantial population size, the number of people with hepatitis A and hepatitis E infection in China is also substantial (6).

There have been many epidemiological studies of viral hepatitis in China, including some that have revealed the temporal trends of different types of viral hepatitis in China (7–9). For instance, Zhang et al. (7) used data from China’s National Notifiable Disease Reporting System and reported that the HBV incidence was stable, the incidence of hepatitis C and E increased and the incidence of hepatitis A decreased from 2004 to 2016. However, previous studies mostly focus on the incidence of viral hepatitis and make little effort to predict further disease burden. A comprehensive temporal analysis of the incidence and mortality of viral hepatitis and their long-term prediction remains scarce in the literature. Understanding patterns and trends in both the incidence and mortality of viral hepatitis and predicting future trends are important for prevention, treatment and guiding resource allocation of viral hepatitis.

Here, we provide a comprehensive temporal analysis of acute viral hepatitis and its type using the most updated data from the Global Burden of Diseases, Injuries, and Risk Factors Study (GBD 2019) to estimate the incidence and mortality of hepatitis from 1990 to 2019 and make predictions to 2030. Our results will provide valuable insights for evidence-based healthcare planning, resource allocation of viral hepatitis control, and prevention in China.

## Materials and Methods

### Data Source

The incidence and mortality from viral hepatitis were obtained from GBD 2019 (10), which provides comprehensive estimates of 315 causes of morbidity and mortality in 195 countries from 1990 to 2019. In short, the GBD study provides a standardized framework for integrating, validating, analyzing, and disseminating the burden of disease and for assessing the burden of premature death, health loss, and disability from disease, injury, and risk factors in diverse populations. The GBD study assembles data from multiple sources, including published systematic reviews and meta-analyses, cohort studies, cross-sectional studies, case reports, Global Health Databases, WHO libraries and WHO regional databases, vital registration databases, sample registration systems, household surveys, censuses and health and demographic surveillance sites. The GBD 2019 used DisMod-MR 2.1, a meta-regression tool, to pool the incidence and mortality data and generate location-year-age-sex-specific estimates. Detailed descriptions of the modeling strategy for incidence and mortality estimation and validation have been published elsewhere (10).

Our study focuses on the incidence and mortality of acute viral hepatitis in China, including acute hepatitis A, hepatitis B, hepatitis C, and hepatitis E. Acute hepatitis A, hepatitis B, hepatitis C and hepatitis E are defined as the presence of anti-HAV IgM, HBsAg for 6 months or fewer, anti-HCV and/or HCV RNA for 6 months or fewer, anti-HEV IgM, respectively. The ICD-10 coding of acute hepatitis A, hepatitis B, hepatitis C, and acute hepatitis E were B15, B16, B17.0, B17.1, and B17.2, respectively. The incidence and mortality data were stratified by age (i.e., 0–14, 15–49, 50–69, and ≥70 years), calendar year (1990–2019), and sex (male and female).

### Statistical Analysis

Trends in viral hepatitis ASIR (reported per 100,000 person-years) and ASMR were plotted using locally weighted regression (LOESS). We used joinpoint regression analysis to detect changes in temporal trends and to estimate the annual percentage change (APC) and corresponding 95% CI for each trend segment (11). We also estimated the average annual percentage change (AAPC) assuming only one homogeneous trend over the entire range of our study period.

### Prediction

Age-period-cohort models are frequently used to describe time trends of incidence or mortality and predict further trends. The age-period-cohort model assumes that the change in incidence or mortality can be attributed to three effects: age, period, and cohort. The projection of incidence rates using age-period-cohort models is of great interest due to demographic changes and because of therapeutic and diagnostic developments (12, 13). We defined prior distributions for age, period, and cohort effects that smooth each point on the two preceding points (random walk 2) in the Bayesian age-period-cohort model. A Bayesian age-period-cohort analysis was employed to describe ASIR and ASMR trends between 1990 and 2019 and projections to 2030.

Bayesian age-period-cohort models incorporate prior information about smoothness on each time scale to reduce random variation and improve the precision of the projections (14). Joinpoint regression was performed using the Joinpoint Regression Program version 4.8.0.1 (Statistical Research and Applications Branch, National Cancer Institute). Other analyses and data visualization were performed using R version 4.0.0 (R Foundation for Statistical Computing, Vienna, Austria).

## Results

### Overall Change in Acute Viral Hepatitis

Table 1 shows the change in incidence and mortality from acute viral hepatitis between 1990 and 2019 in China. In 1990, there were 67 million incident cases of acute viral hepatitis, which then decreased to 47 million incidence cases in 2019. Males have a larger number of incident cases of acute hepatitis than females in China, and the number of incident cases declined between 1990 and 2019 in both men and women. The number of incident cases decreased in all age groups from 1990 to 2019, but the decrease was more pronounced in people aged less than 15 years old. Hepatitis A and B account for the majority of acute viral hepatitis (90.1%) in China, yet the decrease between 1990 and 2019 was most pronounced in hepatitis B and C. The number of deaths from acute viral hepatitis in China is substantially lower than the number of incident cases. There were 26,162 deaths from acute viral hepatitis in 1990 and then decreased to 3,726 in 2019. The patterns of mortality in different genders and age groups were similar to that of incidence, but the decreases were more pronounced for mortality.

**TABLE 1:** Change of incidence and mortality for viral hepatitis between 1990 and 2019 in China.

	Incidence					Mortality				
	1990		2019			1990		2019		
Characteristics	Number	ASR	Number	ASR	Change in rates, %	Number	ASR	Number	ASR	Change in rates, %
Overall	67206390	5506.67	46744682	3768.10	−31.6	26162	2.66	3726	0.20	−92.3
**Gender**										
Male	38603733	6095.02	27402638	4144.76	−32.0	16651	3.35	2661	0.30	−91.1
Female	28602658	4883.08	19342044	3382.12	−30.7	9511	1.99	1065	0.12	−94.1
**Age groups**										
0–14	30018410	9247.02	12860350	5685.54	−38.5	6802	2.07	138	0.06	−96.9
15– 49	32579188	4761.76	25364502	3560.46	−25.2	8006	1.35	836	0.10	−92.6
50–69	3910466	2532.95	7097649	1909.18	−24.6	7566	4.94	1543	0.42	−91.5
70 plus	698327	1768.24	1422180	1295.07	−26.8	3788	10.42	1208	1.17	−88.8
**Specific etiologies**										
HAV	27889161	2301.79	19369254	1960.92	−14.8	12788	1.29	601	0.03	−97.4
HBV	33155300	2699.35	23094874	1384.26	−48.7	11474	1.18	2888	0.16	−86.6
HCV	891619	77.28	478123	47.11	−39.0	1046	0.11	90	0.00	−95.5
HEV	5270311	428.25	3802431	375.81	−12.2	854	0.09	148	0.01	−91.0

*ASR, age-standardized rates; HAV, hepatitis A; HBV, hepatitis B; HCV, hepatitis C; HEV: hepatitis E.*

**FIGURE 1:**
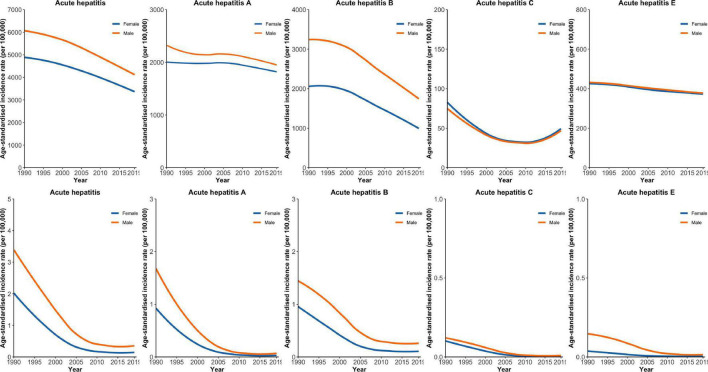
Temporal trends in age-standardized incidence rate (ASIR) and age-standardized mortality rate (ASMR) for total acute viral hepatitis and its type by gender.

Figure 1 presents the temporal trends of the age-standardized incidence rate (ASIR) and age-standardized incidence rate (ASMR) for total acute viral hepatitis and its type by sex. The ASIR of hepatitis B is substantially higher in males than in females. However, there was little gender difference in ASIR for hepatitis A, C, and E. There were declining trends for ASIR of total acute viral hepatitis and its type, and the declining trend was most apparent for hepatitis B. Of note, a U-shaped trend was observed for ASIR of hepatitis C. The ASMR of total acute viral hepatitis and its type were consistently higher in males than in females. We also observed declining trends for ASMR of total acute viral hepatitis and its type, characterized by a rapid decline from 1990 to 2005, followed by a slow decline from 2006 to 2019.

### Results of Joinpoint Regression Analysis

Table 2 presents the results of joinpoint regression analysis for ASIR and ASMR of viral hepatitis in China. The ASIR of acute viral hepatitis showed a persistent decline, with an AAPC of −1.9% (95% CI: −1.9, −1.8) between 1990 and 2019, presenting with three periods, including an APC of −1.3% (95% CI: −1.4, −1.2) between 1990 and 1997, an APC of −2.0% (95% CI: −2.0, −1.9) between 1997 and 2010, and an APC of −2.2% (95% CI: −2.3, −2.1) between 2010 and 2019. Males and females share a similar pattern of decline for ASIR. Overall declining trends were observed for hepatitis A, B, C, and E. Of note, hepatitis C had the most pronounced decline, with an AAPC of −2.6% (95% CI: −3.2, −2.0), which was substantially higher than that of other viral hepatitis types. The trend of ASIR for hepatitis C presented with three periods, and there was a unique recent rapid increase in ASIR with an APC of 10.9% (95% CI: 8.1, 13.8). The joinpoint regression results of ASMR were generally similar to the findings of ASIR, yet the magnitude of decline was larger for ASMR. For etiology-specific viral hepatitis, hepatitis A and hepatitis C had larger declines, with AAPCs of −10.6% (95% CI: −11.1, −10.1) and −9.0% (95% CI: −9.4, −8.5), respectively, than those of other viral hepatitis types.

**TABLE 2:** Joinpoint regression analysis for ASIR and ASMR of viral hepatitis in China, 1990–2019.

	1990–2019		Period 1		Period 2		Period 3	
	Period	AAPC	Period	APC	Period	APC	Period	APC
Total	1990–2019	−1.9 (−1.9, −1.8)	1990–1997	−1.3 (−1.4, −1.2)	1997–2010	−2.0 (−2.0, −1.9)	2010–2019	−2.2 (−2.3, −2.1)
**Sex**								
Male	1990–2019	−1.8 (−1.8, −1.7)	1990–1995	−1.2 (−1.4, −1.1)	1995–2000	−1.4 (−1.7, −1.2)	2000–2019	−2.0 (−2.0, −2.0)
Female	1990–2019	−2.0 (−2.1, −2.0)	1990–1995	−1.2 (−1.4, −1.0)	1995–2010	−2.1 (−2.1, −2.1)	2010–2019	−2.3 (−2.4, −2.2)
**Etiology**								
HAV	1990–2019	−1.9 (−2.0, −1.8)	1990–2001	−2.6 (−2.8, −2.5)	2001–2013	−1.8 (−2.0, −1.7)	2013–2019	−0.8 (−1.2, −0.4)
HBV	1990–2019	−1.9 (−1.9, −1.8)	1990–2000	−0.4 (−0.5, −0.3)	2000–2012	−2.2 (−2.2, −2.1)	2012–2019	−3.4 (−3.5, −3.2)
HCV	1990–2019	−2.6 (−3.2, −2.0)	1990–2004	−8.2 (−8.6, −7.8)	2004–2014	−0.9 (−2.1,0.3)	2014–2019	10.9 (8.1,13.8)
HEV	1990–2019	−1.8 (−1.8, −1.7)	1990–1995	−1.2 (−1.5, −0.9)	1995–2014	−2.2 (−2.2, −2.1)	2014–2019	−0.8 (−1.1, −0.4)
Total	1990–2019	−7.1 (−7.4, −6.7)	1990–1999	−7.4 (−7.8, −7.0)	1999–2007	−13.7 (−14.6, −12.9)	2007–2019	−2.1 (−2.7, −1.5)
**Sex**								
Male	1990–2019	−6.8 (−7.1, −6.4)	1990–2000	−6.8 (−7.1, −6.5)	2000–2006	−15.0 (−16.3, −13.7)	2006–2019	−2.6 (−3.1, −2.2)
Female	1990–2019	−7.9 (−8.3, −7.5)	1990–1998	−8.6 (−9.1, −8.1)	1998–2007	−14.4 (−15.2, −13.6)	2007–2019	−2.2 (−2.9, −1.4)
**Etiology**								
HAV	1990–2019	−10.6 (−11.1, −10.1)	1990–1999	−11.0 (−11.5, −10.6)	1999–2008	−17.0 (−18.0, −16.1)	2008–2019	−4.5 (−5.6, −3.4)
HBV	1990–2019	−5.3 (−5.6, −4.9)	1990–2000	−5.0 (−5.4, −4.7)	2000–2006	−13.6 (−15.0, −12.3)	2006–2019	−1.3 (−1.8, −0.8)
HCV	1990–2019	−9.0 (−9.4, −8.5)	1990–1999	−6.1 (−6.6, −5.7)	1999–2009	−15.8 (−16.5, −15.1)	2009–2019	−4.2 (−5.4, −3.1)
HEV	1990–2019	−6.9 (−7.5, −6.4)	1990–1999	−4.1 (−4.8, −3.5)	1999–2008	−12.8 (−13.8, −11.7)	2008–2019	−4.3 (−5.3, −3.2)

*ASIR, age-standardized incidence rates; ASMR, age-standardized mortality rates; AAPC, average annual percent of change; APC, estimated annual percent of change; HAV, hepatitis A; HBV, hepatitis B; HCV, hepatitis C; HEV, Hepatitis E.*

### Predictions for Acute Viral Hepatitis

**FIGURE 2:**
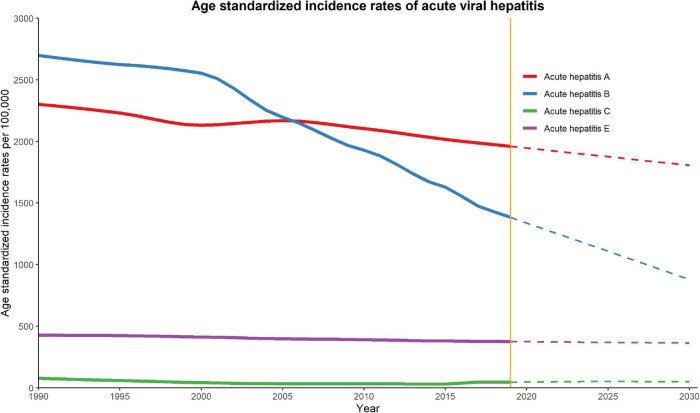
Trends in observed (solid lines) and predicted (dashed lines) age-standardized incidence rate (ASIR) of acute viral hepatitis from 1990 to 2030 in China.

**FIGURE 3:**
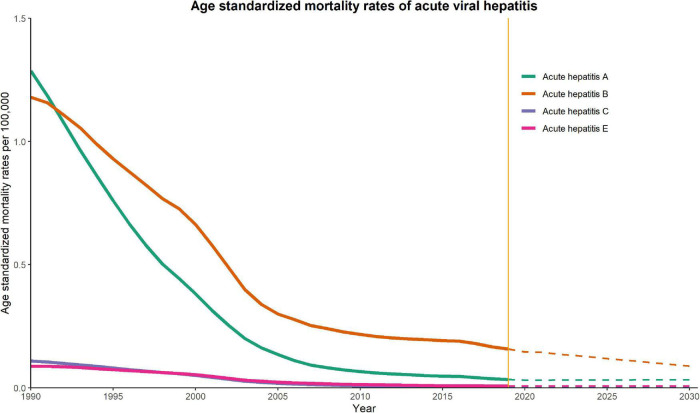
Trends in observed (solid lines) and predicted (dashed lines) age-standardized mortality rate (ASMR) of acute viral hepatitis from 1990 to 2030 in China.

Figures 2, 3 show the observed and predicted trends for ASIR and ASMR of acute viral hepatitis from 1990 to 2030 in China. The ASIRs of hepatitis A and hepatitis B are substantially higher than those of other hepatitis types. We observed that hepatitis B had the most substantial decline in ASIR between 1990 and 2019, and this rapid decline was predicted to continue and reach approximately 1000 per 100,000 in 2030. The ASIR of hepatitis A also declined in the observation and prediction period, but the decline was less than that of hepatitis B. The ASIRs of hepatitis C and E were generally low and were predicted to be stable until 2030. The ASMR of hepatitis A and hepatitis B was substantially higher than that of other hepatitis types, and both demonstrated a rapid decline between 1990 and 2005 followed by a slow decline until 2030. Our prediction results indicate that the ASMR of hepatitis A, B, C, and E are all very low.

## Discussion

This analysis shows generally favorable decreasing trends for both the incidence and mortality of viral hepatitis in China. However, there are also variations in those trends among different types. Of note, despite the overall decline in the incidence of hepatitis C between 1990 and 2019, there was a very alarming increase in hepatitis C incidence from 2014 to 2019 in China. The mortality due to acute viral hepatitis is low; however, the absolute number of deaths from viral hepatitis remains high in China, and some types of viral hepatitis including hepatitis B and C may progress to chronic infections, which will cause long-term complications such as cirrhosis and liver cancer. Despite the overall decline of both the incidence and mortality of viral hepatitis in China, the disease burden of viral hepatitis remains high, which underscore the importance of continued public health interventions for viral hepatitis prevention.

China is the country most affected by hepatitis B in the world, and the elimination of hepatitis B in China will be a major contributor to the global elimination of hepatitis B by 2030 (15). Hepatitis B is one of the most important infectious diseases in China. According to the 1992 National Serological Survey, the prevalence of HBsAg infection in the Chinese population was 9.8% in all age groups and then declined to 7.2% in the population aged 1–59 years in 2006, while the prevalence in children under 5 years was only 1% (16). Our findings further confirm the decreasing trend for hepatitis B incidence, with a −48.7% decrease from 1990 to 2019. In addition, our joinpoint analysis revealed that despite the overall decrease in hepatitis B incidence, the most rapid decline periods started in 2000, which coincided with the timeline of China’s national hepatitis B immunization program. Although the hepatitis B vaccine was recommended for infant routine immunization in 1992 in China, until 2002, the hepatitis B vaccine was fully integrated into infant routine immunization, and all vaccines and administration fees were fully paid by the government (17). In addition, there was a catch-up campaign for children younger than 15 years to ensure hepatitis B vaccine coverage in rural areas from 2009 to 2011. Since then, there has been a steady increase in hepatitis B vaccine coverage, and the reported coverage of three doses of hepatitis B for infants has increased from 30.0% in 1992 to 99.6% in 2015 (18). With this high coverage of vaccines, it is anticipated that the hepatitis B incidence will continue to decrease.

Hepatitis A is another important viral hepatitis concern in China. Our analysis revealed that the incidence of hepatitis A decreased by 14.8%, but the mortality decreased by 97.4% from 1990 to 2019. The most common transmission model of hepatitis A outbreaks in China is the use of contaminated food and water (19, 20). The most effective way to prevent hepatitis A infection is vaccination, which was first available in China in 1992. Unlike the hepatitis B vaccine, before 2008, the hepatitis A vaccine was a class 2 vaccine, which was not routinely recommended for childhood vaccination and only available to persons willing to pay for vaccination (21). Therefore, the decline in hepatitis A incidence was not as good as hepatitis B in China. However, with the steady increase in hepatitis A vaccine coverage, the hepatitis A incidence in China will be further reduced. Although there is no specific treatment for hepatitis A, decreasing hepatitis A mortality may require the early identification of hepatitis A and maintaining comfort and adequate nutritional balance. Therefore, the well-established National Notifiable Infectious Disease Surveillance System in China and the increased access to healthcare may explain the substantial reduction in hepatitis A mortality.

Our study reveals a recent increase in hepatitis C incidence rates in China. This finding was consistent with the results reported by other studies (22, 23). However, it is noted that this increase in hepatitis C incidence rates in China does not necessarily reflect the increased risk of hepatitis C in the Chinese population but may be due to the improved diagnosis of hepatitis C in China (24). Our prediction analysis also indicates that the AMIR for hepatitis C will be stable at a low level through 2030. The overall burden of hepatitis C in China is substantial because of its giant population size (25). This increased hepatitis C incidence may cause a potential threat to the public health of China. Unlike other viral hepatitis diseases, such as hepatitis A, B or even E, there is no safe and effective vaccine to prevent hepatitis C (26). Therefore, behavioral interventions are needed to constrain the spread of hepatitis C in China. Fortunately, there is tremendous progress in hepatitis C treatment, and new direct-acting antiviral agents (DAAs) may achieve a sustained virologic response (SVR) in HCV-infected people as high as 97%(27).

The number of deaths from acute viral hepatitis A, B, C, and E was generally substantially lower than the number of incident cases. However, hepatitis A and E cause only acute hepatitis, but hepatitis B and C may cause long-term complications, including cirrhosis, liver cancer, and even death (28, 29). Although the mortality from acute viral hepatitis B and C is low in China, the long-term mortality from chronic hepatitis B and C is huge given the larger number of people infected in China. The primary prevention of hepatitis B and C may prevent long-term complications and mortality. However, for people who have been infected with hepatitis B and C, timely diagnosis and treatment of hepatitis B and C may substantially reduce the possibility of related complications and long-term mortality (30), which is especially important for hepatitis C because DAAs may cure over 96% of infected people. The development of hepatitis B treatment is suboptimal compared with hepatitis C treatment. Current nucleos(t)ide therapy for hepatitis B can lead to HBsAg negativation of only 1–3% per year (31, 32), but the treatment can also substantially reduce the possibility of disease progression of hepatitis B.

### Limitations

Several limitations should be acknowledged before interpreting the results. First, all the data used in our study are from the 2019 GBD study, which collected data from different sources. Therefore, inconsistencies and incompatibilities between these data might exist, which may cause bias. Second, the GBD study does not provide site-specific data within China; therefore, it is impossible to investigate the geographic pattern of acute viral hepatitis in China. Finally, our prediction was based on the Bayesian age-period-cohort model, which only models the change due to age, period, and cohort effect given the current situation. Any potential treatment diagnosis in the future was not taken into account.

## Conclusion

Our study reveals favorable declining trends of incidence and mortality for acute viral hepatitis in China from 1990 and 2019, and these favorable trends are predicted to continue up to 2030. There are several implications from our findings. Firstly, the decrease in hepatitis A and B incidence may because of the expansion of vaccines in China. However, the absolute number of hepatitis A and B remains substantial in China. A scaled-up vaccine campaign is still needed to tackle the large number of those vaccine preventable hepatitis infections. Secondly, there was a recent upward trend in the incidence of hepatitis C, which is alarming and warrants further close surveillance. Lastly, hepatitis B and C may cause long-term complications, timely diagnosis and treatment of hepatitis B and C may reduce the long-term disease burden caused by hepatitis B and C.

## Data Availability Statement

The original contributions presented in the study are included in the article/supplementary material, further inquiries can be directed to the corresponding author.

## Author Contributions

XS and XH: conceptualization. XS, LZ, TS, and XH: data curation. XS, LZ, and HZ: formal analysis and visualization. LZ and HZ: project administration. XH: supervision. XS, YL, and XH: writing – original draft. All authors: final approval of manuscript.

## Conflict of Interest

The authors declare that the research was conducted in the absence of any commercial or financial relationships that could be construed as a potential conflict of interest.

## Publisher’s Note

All claims expressed in this article are solely those of the authors and do not necessarily represent those of their affiliated organizations, or those of the publisher, the editors and the reviewers. Any product that may be evaluated in this article, or claim that may be made by its manufacturer, is not guaranteed or endorsed by the publisher.

## Abbreviations

ASIR: age-standardized incidence rate
ASMR: age-standardized mortality rate
LOESS: locally weighted regression
APC: annual percent of change
CI: annual percent of change
AAPC: average annual percent of change
GBD 2019: Global Burden of Diseases Injuries and Risk Factors Study
DAAs: direct-acting antiviral agents
SVR: sustained virologic response.
